# Human Mesenchymal Cells from Adipose Tissue Deposit Laminin and Promote Regeneration of Injured Spinal Cord in Rats

**DOI:** 10.1371/journal.pone.0096020

**Published:** 2014-05-15

**Authors:** Karla Menezes, Marcos Assis Nascimento, Juliana Pena Gonçalves, Aline Silva Cruz, Daiana Vieira Lopes, Bianca Curzio, Martin Bonamino, João Ricardo Lacerda de Menezes, Radovan Borojevic, Maria Isabel Doria Rossi, Tatiana Coelho-Sampaio

**Affiliations:** 1 Institute of Biomedical Sciences, Federal University of Rio de Janeiro, Rio de Janeiro, Rio de Janeiro, Brazil; 2 Institute of Biophysics Carlos Chagas Filho, Federal University of Rio de Janeiro, Rio de Janeiro, Rio de Janeiro, Brazil; 3 National Institute of Cancer, Rio de Janeiro, Rio de Janeiro, Brazil; 4 Excellion, Petrópolis, Rio de Janeiro, Brazil; National Institutes of Health, United States of America

## Abstract

Cell therapy is a promising strategy to pursue the unmet need for treatment of spinal cord injury (SCI). Although several studies have shown that adult mesenchymal cells contribute to improve the outcomes of SCI, a descripton of the pro-regenerative events triggered by these cells is still lacking. Here we investigated the regenerative properties of human adipose tissue derived stromal cells (hADSCs) in a rat model of spinal cord compression. Cells were delivered directly into the spinal parenchyma immediately after injury. Human ADSCs promoted functional recovery, tissue preservation, and axonal regeneration. Analysis of the cord tissue showed an abundant deposition of laminin of human origin at the lesion site and spinal midline; the appearance of cell clusters composed of neural precursors in the areas of laminin deposition, and the appearance of blood vessels with separated basement membranes along the spinal axis. These effects were also observed after injection of hADSCs into non-injured spinal cord. Considering that laminin is a well-known inducer of axonal growth, as well a component of the extracellular matrix associated to neural progenitors, we propose that it can be the paracrine factor mediating the pro-regenerative effects of hADSCs in spinal cord injury.

## Introduction

Traumatic brain and spinal cord injuries affect individuals of all ages, causing various degrees of disability [1], [2]. Despite intensive research over the last decade aiming to develop new cell-based therapies to treat trauma in the Central Nervous System (CNS), there is no consensus about the most appropriate cell types to reach this goal [3], [4]. Embryonic stem cells previously differentiated into neural precursors, motor neurons, or pre-oligodendrocytes have been used in animal studies [5]–[8] and more recently, in a clinical study [9]. Adult stem/progenitor cells have also been used in both animal research and clinical studies. The primary source of progenitor cells is the bone marrow, where at least the hematopoietic stem cell and a population of mesenchymal stromal/stem cells co-exist (MSC) [10]. Both cell types have been used to treat experimental spinal cord injury (SCI) in animals, as well as injured human patients [11]. Given the potential of MSC to differentiate into several adult cell types, including neurons [12], these cells have attracted great interest.

In the last years, a growing number of studies have reported the effects of MSCs in promoting functional improvement, tissue sparing, and axonal growth after spine cord injury [13]–[23]. More recently, a new type of MSC isolated from adipose tissue has been investigated. Adipose tissue constitutes a more readily available deposit of adult progenitors due to its greater abundance of MSC-like cells, if compared to bone marrow, and because liposuction is a minimally invasive procedure. Known as adipose-derived stromal cells (ADSCs), these cells are isolated by selective adhesion and correspond to the perivascular stromal fraction of the adipose tissue [10], [24], [25]. ADSCs have recently been used to treat SCI in rats and dogs [26]–[28]. In particular, a recent study compared the effectiveness of hADSCs with that of human bone marrow stromal cells in a model of section injury in immunossupressed rats and reported the superior regenerative effect of hADSCs [29].

In the present study we investigated the regenerative potential of hADSCs in a model of ballon-induced spinal compression using immunocompetent rats. We show that hADSCs promote complete recovery of motor function after 8 weeks, while improving tissue preservation, restricting inflammation and stimulating axonal growth. In addition we propose that the regenerative effects of hADSCs are related to the secretion of the extracellular matrix protein laminin that accumulates in the spinal cord in colocalization with neural precursors.

## Results

### hADSC Promotes Functional and Morphological Recovery after Compressive SCI

**Figure 1:**
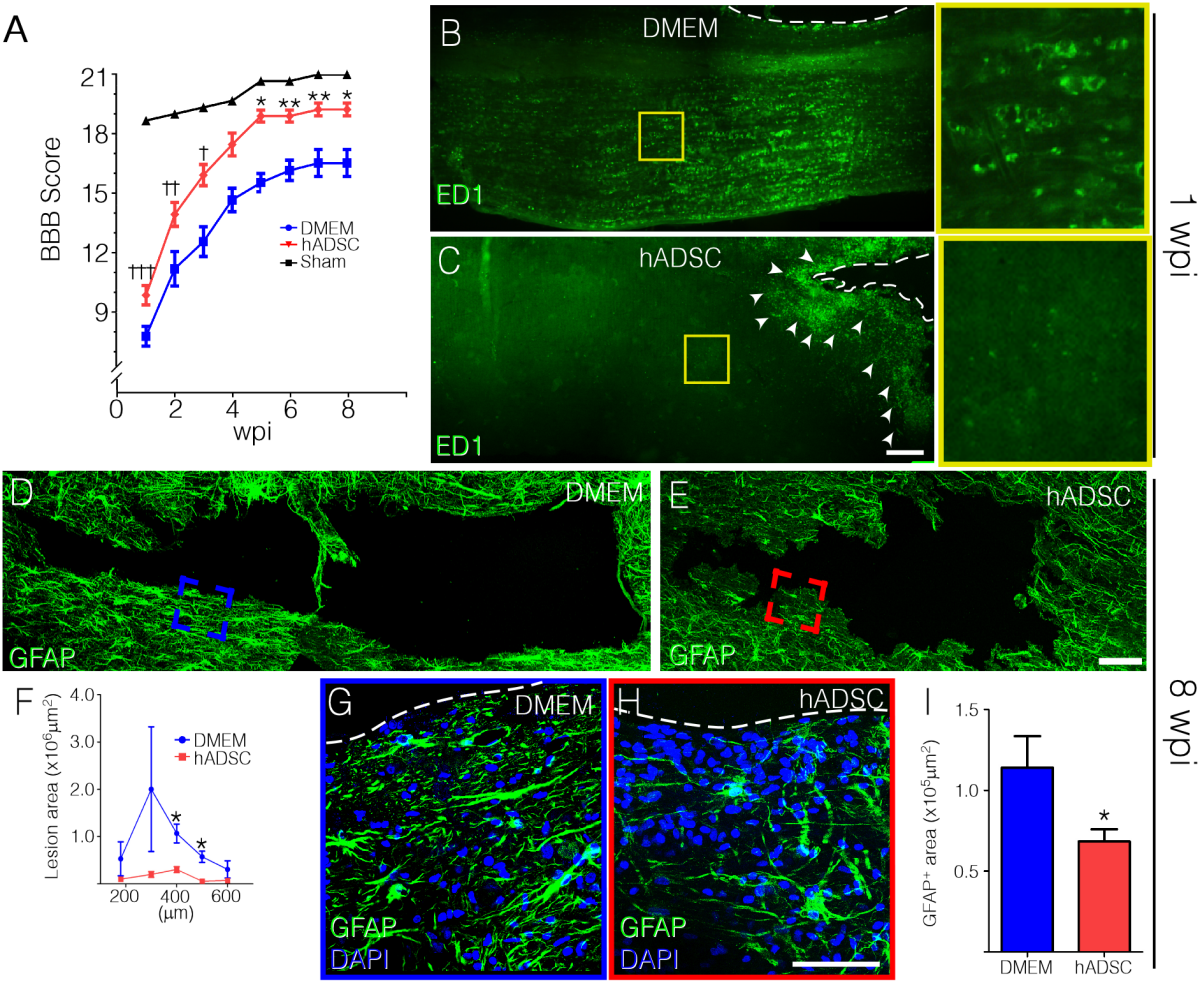
hADSCs induce functional recovery and reduce cavitation and cellular inflammatory response. The locomotor performance was assessed by the BBB score. Animals were evaluated weekly, during eight weeks after spinal compression (A). Asterisks indicate statistic differences between hADSC (n = 9) and DMEM groups (n = 9) and crosses indicate differences between hADSC and sham groups (n = 3). B,C) Low-magnification photomontages showing the marked difference in distribution of macrophages in the spinal cord of rats treated with hADSC (C) and DMEM (B). Note that ED1-positive cells concentrate in the immediate surroundings of the lesion cavity in hADSC-treated animals (arrowheads), in contrast to the dispersed distribution in DMEM-treated animals. D, E) GFAP immunoreactivity delineates the cavity and reveals astrocyte activation following injury, in vehicle (DMEM) (D) and hADSC (E) treated animals. F) Graph representing the area of the lesion cavity of 5 different dorso–ventral sections (represented in the x axis) in DMEM (blue) and hADSC (red) treated animals (n = 3 per condition). Note that cavitation in hADSC-treated animals is smaller. G,H) High magnification images of the boxed areas in *D* (blue) and *E* (red) are shown in *G* and H, respectively. Note the few GFAP-positive cells at the vicinity of the lesion and that very few cells are seen at the cavity border in hADSC-treated rat (H). I) Graph representing the total area of positive staining for GFAP (see *Materials and Methods*) in the immediate surroundings of the cavity in DMEM (blue) and hADSC (red) treated animals (n = 3 per condition). Dashed line delineates the cavity borders in G and H. *P<0.05, **P<0.01, ***: p<0.001. Bars: B and C = 500 µm, D and E = 100 µm and G and H = 50 µm.

In order to evaluate if human subcutaneous ADSCs (hADSCs) would improve the functional outcome after compressive SCI, we compared the open field locomotion (BBB scores) of animals receiving cells or culture medium (DMEM). Rats were subjected to moderate balloon compression and treated with either hADSCs or DMEM, delivered by intraspinal injection 30 minutes after injury. The BBB score for the DMEM group was 7.2 one week post injury/injection (wpi) and it increased to 15.6 after eight weeks (Fig. 1A). On the other hand, animals treated with hADSCs exhibited superior scores from the first evaluation, whereas such superiority became statistically significant from the fifth week on. From the fourth week on the BBB scores for the treated group were indistinguishable from those found for the sham operated group (Fig. 1A), indicating that hADSCs promoted complete functional recovery after a moderate compressive injury.

Mesenchymal stromal/stem cells are known to have immunosuppressive properties [30], [31], enabling the use of human cells in immunocompetent animals as done in this study. We evaluated the distribution of the rat macrophages/microglia marker, ED1 in the spinal cord one week after compression. In the control animals, macrophages/microglia were profusely distributed in both white and gray matter (Fig. 1B). In animals receiving hADSC, macrophages were concentrated in lesion borders (Fig. 1C), suggesting that the injection of hADSC restrained the spread of inflammatory cells in the spinal cord parenchyma.

The compression injury led to the formation of cystic cavities in the spinal cord. Eight weeks after injury, control animals showed a large cavity surrounded by activated astrocytes (Fig. 1D, G). Animals receiving hADSC displayed smaller cavities and a reduction of GFAP expression of approximately 50% (Fig. 1E, H, I). Quantitative analysis of the cavity area was performed in a region encompassing 400 µm of the spinal cord tissue along the dorsal–ventral axis (Fig. 1F). Treatment with hADSCs reduced the cavity area by more than 90% at the lesion epicenter, whereas tissue sparing was seen at all levels analyzed (Fig. 1F). These results demonstrate that hADSC injection results in improved preservation of the nervous tissue after SCI.

### hADSC Promotes Regeneration of Axonal Fibers

**Figure 2:**
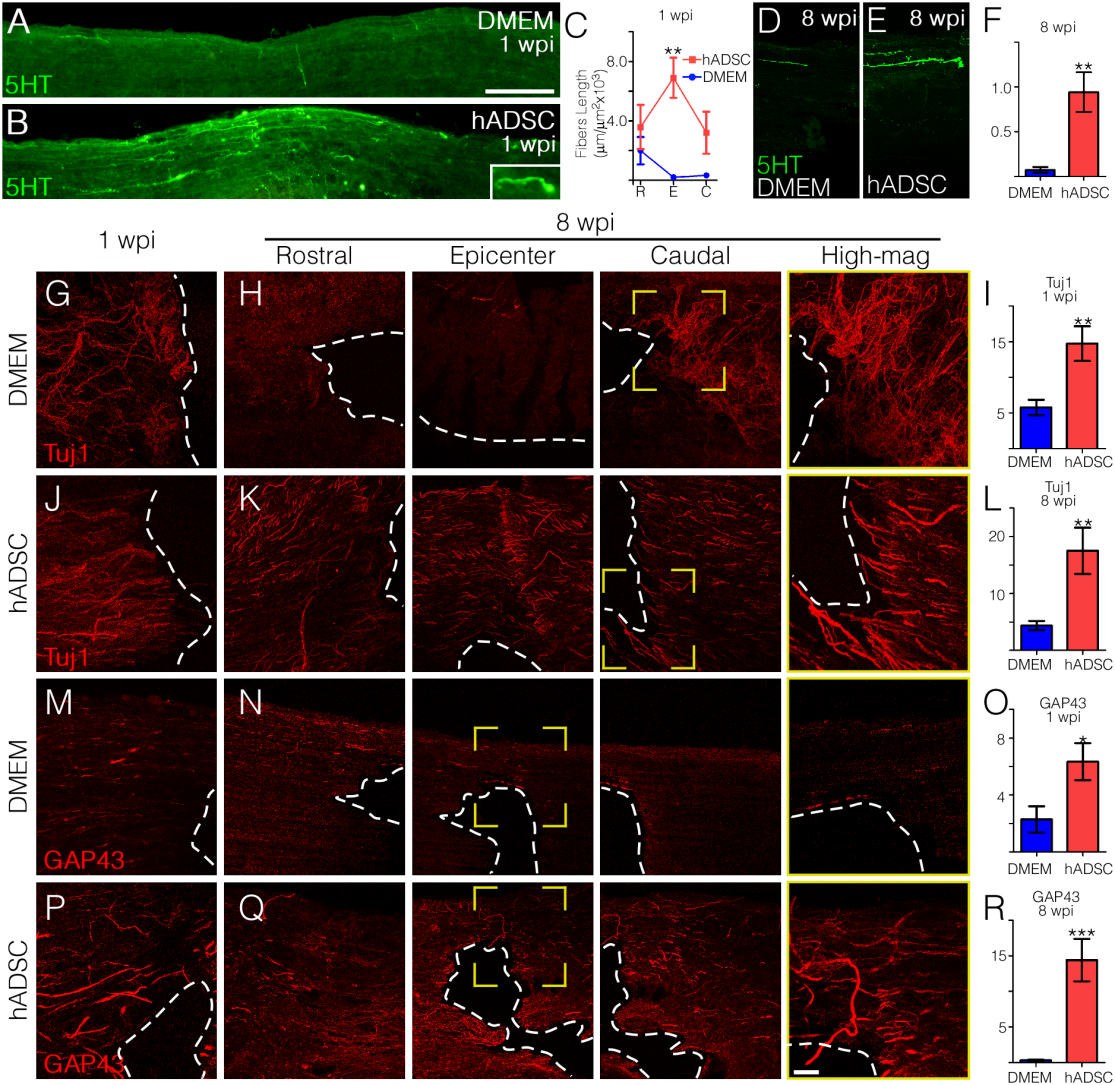
hADSCs promote axonal regeneration. A–D) Photomontages showing the expression pattern of 5HT-positive serotonergic fibers in horizontal sections spanning the pre- and post-lesion regions in DMEM (A) and hADSC (B) treated animals one week after compression. The inset shows an example of a hogback termination in the post-lesion area. Note that the hADSC-treated cord exhibits a larger number of serotoninergic fibers crossing the injury site. C) Graph representing the total length of 5-HT fibers in each experimental group (n = 3 in each group). D–E) 5HT expression was greatly reduced in the chronic phase (8 wks) in DMEM-treated rats (D), while it was slightly reduced in hADSC (E) treated group. F) Quantification of the total length of 5-HT positive axons, eight weeks after SCI (n = 3 in each group). G–R) Progression of axonal regeneration was followed by Tuj1 (G–L) or anti-GAP-43 (M–R) immunoreactivity. Panels on the left show Tuj1 (G, J) or GAP-43 (M, P) positive fibers one week after compression at the epicenter of the lesion. Panels H, K, N, Q and rows on the right show respective rostral (left), epicenter (center), and caudal (right) regions of the same section eight weeks after SCI. Far column on the right (yellow frame) shows boxed areas in higher magnification. The dashed line indicates the border of the lesion cavity. Graphics show the quantification of the total lengths of Tuj1 (I, L) or GAP-43 (O, R) positive fibers one week (I, O) and eight weeks (L, R) after SCI in the peri-cavity region (n = 3 in each group). Tuj1 and GAP-43 positive axons in DMEM (blue) and hADSC (red) treated groups were quantified in an area of approximately 900 µm in extension along the longitudinal axis. Both white and grey matter was analyzed. The y-axes in panels F, I, L, O and R were omitted for aesthetic purpose and they represent fiber length per area (µm/µm^2^×10^3^). The values in C, F, I, L, O, R represent means ± standard errors. *P<0.05, **P<0.01, ***P<0.001. Bars: A, B = 500 µm, D–Q = 100 µm, high magnification = 50 µm.

The presence of descending serotonergic fibers was evaluated in animals one and eight weeks after injury. One week after injury, hADSC-treated rats presented a large number of serotonergic fibers crossing the spared tissue in the lateral funiculus region surrounding the injury site (Fig. 2B). These fibers, which were less abundant in control animals, were apparently thicker than individual axons (Fig. 2A). Their superficial location corresponds to the region of descending serotonergic raphe fibers, indicating that they result from axonal regrowth after injury. In addition, seven days after injury we found several fibers displaying bulging tips, which are indicative of the occurrence of axonal regeneration (Fig. 2B, insert). Rostral to the lesion site the total lengths of serotonergic axons were similar in treated and non-treated rats (Fig. 2C). However, at the level of the epicenter (lateral to it) and caudal to the lesion, the total lengths of serotonergic axons were consistently higher after treatment with hADSC. Eight weeks after injury the fiber lengths decreased in comparison to those found one week after injury (Fig. 2D, E). Nevertheless, the total length of serotonergic fibers in the spinal cord was 8 times higher in the group treated with hADSCs (Fig. 2F). These results indicate that the regeneration of serotonergic axons promoted by hADSCs remains for a considerable time.

Axonal regeneration was further evaluated by using either the Tuj1 antibody, which stains young neurons more intensely [32], or anti-GAP-43, which labels neurons in regeneration [33]. Data are presented in series of three pictures taken from the spare tissue lateral to the lesion area. As shown in Figure 2G–R, the treatment with hADSCs substantially increased axonal regeneration. At both 1 and 8 weeks Tuj1-positive fibers in hADSC-treated animals ran straight and were aligned parallel to each other (Fig. 2J and high magnification in K). On the other hand, in control animals these fibers were tangled and randomly aligned (Fig. 2G and high magnification in H). The thicker bundles and aligned fibers observed in hADSC-treated animals are indicative of axonal fasciculation, which did not occur in control animals (Fig. 2, high magnifications in H and K). Tuj1 labeling presented a very faint and even distribution in normal unlesioned animals as this monoclonal antibody identifies preferentially young neurons [32]. Quantification of Tuj1 labeled fibers after one or eight weeks showed that treatment with hADSC led to an increase in the total length of young fibers of 3 or 3.5 times, respectively. Panels M to Q in Figure 2 show the results obtained by using the anti-GAP-43 antibody. One week after injury the total length of regenerating fibers increased 3-fold in the hADSC group relative to the control (Fig. 2M, P, O). Eight weeks after SCI, a large number of GAP-43 positive fibers was observed in rats treated with hADSCs, while control animals showed little staining (Fig. 2N, Q, R). No labeling was detected when anti-GAP43 was used in sections of normal unlesioned rats.

### hADSCs Induce Foci of Increased Cellularity in Spinal Cord

**Figure 3:**
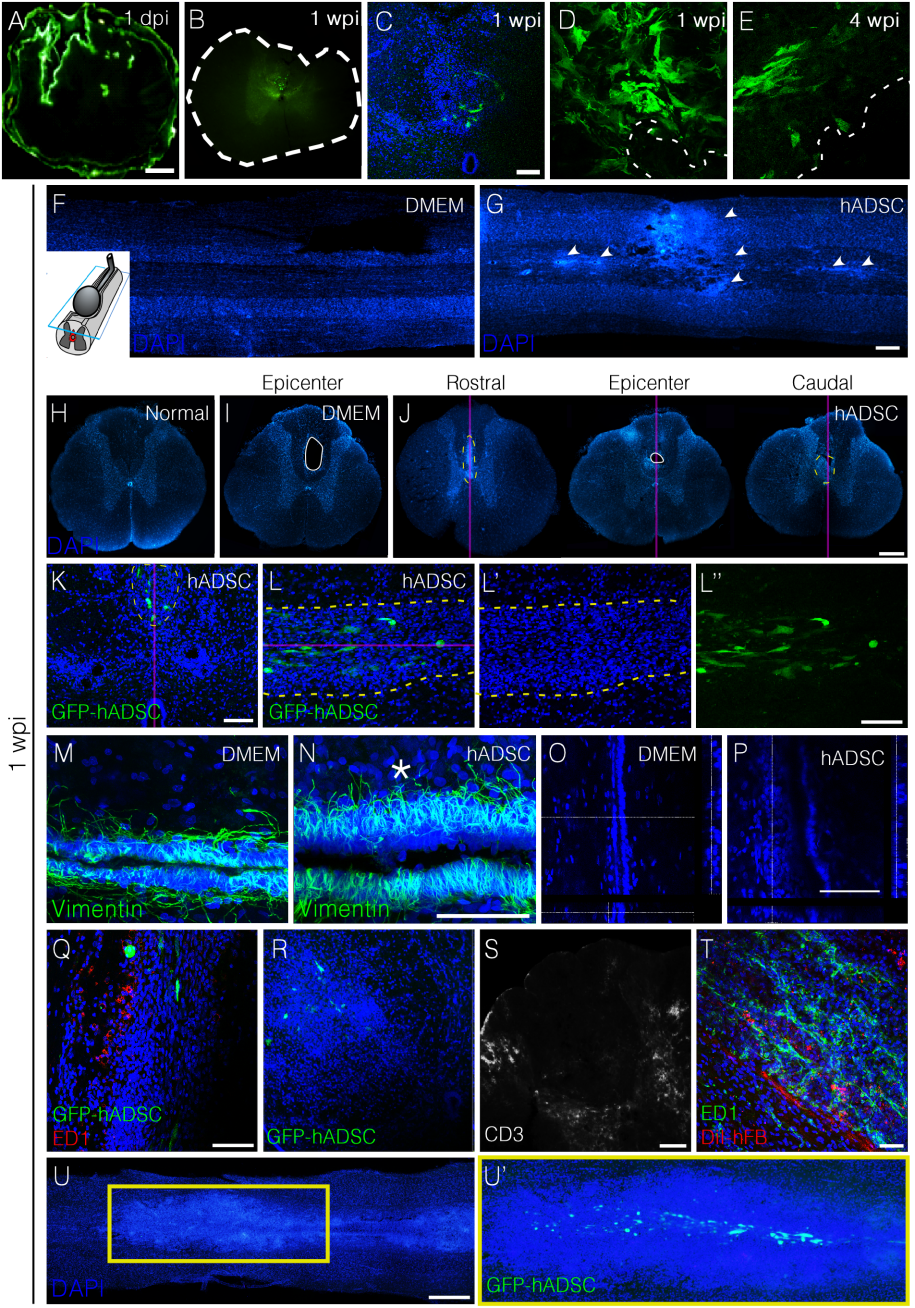
hADSCs increased cellularity in three distinct, but contiguous compartments (the central canal, midline parenchyma, and perivascular region). A–E) Time-course distribution and cell morphology after injection of hADSCs. A) Transverse section of the spinal cord one day after lesion and injection of GFP-transduced hADSCs, showing that these cells accumulate in the pial surface, central canal, and peri-lesional area. After one week (1 wpi), hADSCs were detected predominantly in the canal area and peri-lesionally (B, C). D, E) High magnification images of the GFP-positive cells clusters at one (D) and four wpi (E). F, G) DAPI-stained horizontal sections 200–250 µm above the central canal showing an increase in cellularity (G) mainly along the midline in hADSC-treated cord (arrowheads). H–J) DAPI-stained transversal sections of spinal cord from a normal rat (H), DMEM (I) and hADSCs–treated animals (J), 1 wpi. Sequential analysis of histological sections from treated animals (J) showed that initially there is an increased cellularity along the midline, crossing the region of the central canal of the spinal cord. In the following sections this cell cluster is observed at the margin of the lesion and persisted there until the end of the lesion. K–L”) Confocal images of transversal (K) and horizontal (L, L’, L”) sections of spinal cord of hADSC-treated animal at 1 wpi. Note that the increased cellularity occurs in the region around the GFP-positive cells. M–P) Confocal images of horizontal sections of spinal cord immunostained with anti-vimentin in the central canal at 1 wpi. Note the increased thickness of the ependymal lining of the central canal, and the high cell density in hADSC-treated animal above the canal (asterisk). O, P) Orthogonal views of DAPI-stained section of the central canal. Q–S) Images showing infiltration of macrophages (ED1, panel Q) or T lymphocyte (CD3+ cells, panels R and S). T) Human fibroblasts (stained with CM-DiI, hFB) 1 week post injection into an intact spinal cord. Note the abundance of ED1-positive cells in hFB-treated rat. Confocal images of the cells cluster one week after injection of the hADSC-GFP positive cells into an uninjured spinal cord. The purple line represents the midline of the spinal cord. Bars: A, E, F, H–J = 500 µm; B–D, T = 50 µm; G = 25 µm, K. L, Q, R, S = 100 µm, U = 1 mm.

We next sought to investigate the mechanisms by which hADSCs exert regenerative effects in the spinal cord. GFP-transduced hADSCs were injected 30 minutes after compression and animals were euthanized at different times. One day after injection, cells were densely packed and accumulated mainly in the meningeal surface, the central canal, and at the lesion site (Fig. 3A). One week later, the injected cells were detected at the region of the canal and at the injury site (Fig. 3B–D). In the fourth week, only a few isolated clusters of cells were identified near the lesion (Fig. 3E), while cells were not detected at the eighth week after injection.

A remarkable feature of hADSC-treated animals was the increase in tissue cellularity detected in DAPI-stained sections one week after cell injection. One large cluster of cells was found at the lesion site and smaller clusters were distributed along the spinal midline (Fig. 3G). Such clusters were not observed in normal unlesioned rats (Fig. 3F). In transversal sections it was possible to localize the clusters at the midline in the vicinity of the central canal (Fig. 3I–K). GFP-hADSCs were found in the center of these areas of high cellularity (Fig. 3K, L). Analysis of the section containing the central canal revealed that the vimentin-positive ependymal cell layer was enlarged in animals treated with hADSCs (compare Fig. 3M and N). Reconstruction of the central canal in the z axis showed regions with more than 10 stacked cells, indicative of a partial reconfiguration of the canal’s architecture (Fig. 3P). We additionally observed an increased number of DAPI-stained nuclei throughout the spinal parenchyma, in the region between the central canal and the individual clusters (Fig. 3N, asterisk) and blood vessels in the vicinity of the reconfigured canal (Fig. S1).

Cell clusters did not correspond to inflammatory infiltrates given that most of them were not positive for the macrophage/microglia marker, CD68 (Fig. 3Q), and neither for the T lymphocyte marker, CD3 (Fig. 3R, S). Scattered macrophages were detected in the vicinity of the clusters, as well as T lymphocytes, which tended to form small aggregates surrounding the core of the DAPI-stained clusters. In contrast, DiI-labeled human fibroblasts injected into the intact rat spinal cord attracted large amounts of macrophage/microglial cells (Fig. 3T).

Since the increase in tissue cellularity was not related to injury-induced inflammation, we next investigated whether it would also occur upon injection of the cells into the undamaged spinal cord. We therefore included a third experimental group in which GFP-hADSCs were delivered directly into the spinal cord of normal rats and their distribution was analyzed one week after injection. Cells presented a clear tropism for the spinal midline, where they remained distributed along the central axis of the spinal cord (Fig. 3U’). It was noteworthy that more GFP positive cells were present in uninjured than in the injured spinal cord. Surprisingly we observed a large increase in tissue cellularity, which was more pronounced than that observed when hADSCs were injected after injury (Fig. 3U). Cells within the infiltrate in uninjured spinal cord did not stain for the CD68, which indicate that they were not inflammatory cells (Fig. S2).

### hADSCs Led to the Appearance of Perivascular Spaces in between Endothelial and Astrocytic Basement Membranes

**Figure 4:**
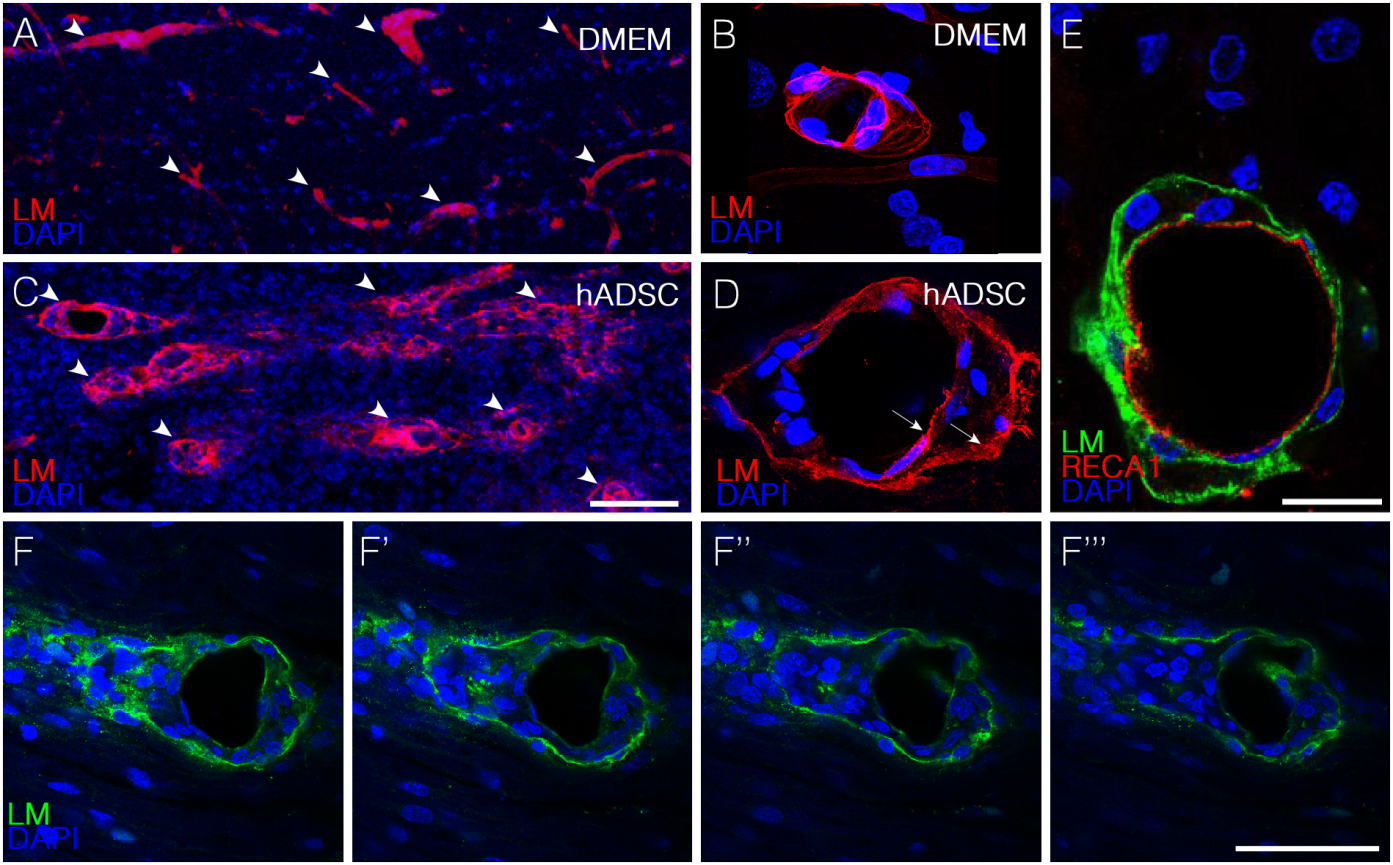
hADSCs led to the appearance of perivascular spaces in between endothelial and astrocytic basement membranes one week after injection. A–D) Confocal images of horizontal sections immunostained with anti-pan-laminin antibody (red) one week after injury. Note that in DMEM animals (A,B) there is no separation between the two membranes whereas in hADSCs–treated animals (C,D) these membranes are separated (arrows in D). E) Confocal images of a horizontal section immunostained with anti-pan-laminin (green) and RECA-1(red). F–F”) Confocal imagens of sequential optical sections immunostained with anti-pan-laminin (green) and DAPI (blue) showing the extravasation of cells from the blood vessels. Bars: C, F = 50 µm B, D, E = 25 µm.

Another remarkable feature observed in animals treated with hADSCs was the presence, in the vicinity of the spinal axis, of blood vessels exhibiting two clearly definable separated basal lamina, which were identified by immunostaining with an anti-pan-laminin antibody (Fig. 4C, D, Movie S1). The internal lamina corresponds to the basement membrane contributed by the endothelial cells, here identified by immunostaining with the rat endothelial marker, RECA1 (Fig. 4E). The external lamina, which is part of the blood brain barrier in the CNS, is produced by the astrocytes and normally remains connected to the internal one. The separation of these two membranes suggests an increased traffic of cells in the perivascular compartment along blood vessels. A series of confocal images of a blood vessel suggests that cells accumulating in between the two laminas can be released at the spinal parenchyma upon disruption of the outer membrane (Fig. 4F). Separation of two basement membranes around blood vessels was also observed when hADSCs were injected into the undamaged spinal cord (Fig. S2).

### Cells within the Infiltrates Express Markers of Neural Precursors

**Figure 5:**
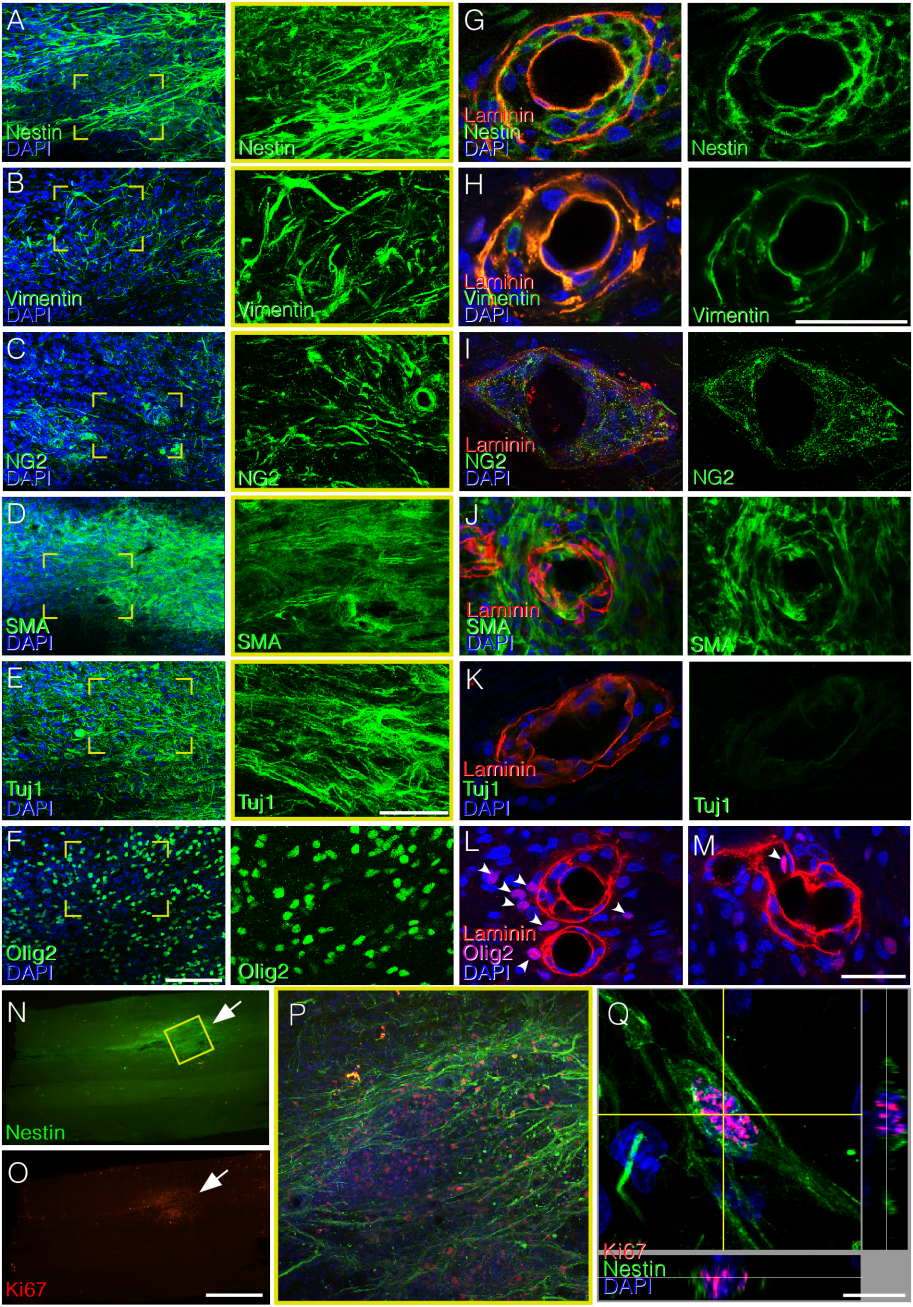
Areas of increased cellularity induced by hADSC are mainly composed of neural precursors, but cell types differ in the vascular and parenchymal compartments. A–F) Confocal images of horizontal sections of the spinal cord showing immunostaining for nestin (A), vimentin (B), NG2 (C), smooth muscle α-actin (αSMA, D), Tuj1 (E), and Olig2 (F) in clusters of high cell density along the midline, one week after SCI. Note that cells in the clusters are positive for all of these markers, suggesting that clusters are mostly composed of neural progenitors and possibly of invading pericytes. Panels on the right correspond to high-magnification images of the boxed areas, showing the labeling for each phenotypic marker. G–M) Blood vessels immunostained with pan-laminin (red) and nestin (G), vimentin (H), NG2 (I), αSMA (J), Tuj1 (K) (green). Panels L and M show that most Olig2 positive cells (purple) are out of blood vessels (L). Panel M depicts one Olig2-labeled nucleus in close proximity with a region where the basal lamina seems to be disrupted (arrow). (N–Q) Correspondence between immunoreactivities for nestin (N) and Ki67 (O) in photomontages of horizontal sections of an injured spinal cord one week after injury/injection. Confocal images showing proliferative activity (anti-Ki67, red) in the spinal parenchyma (P) and a perivascular region (Q) of nestin positive cells. Bars: A–F = 100 µm; G–M = 50 µm; N, O = 1 mm, Q = 10 µm.

In order to indentify the nature of the cells accumulating in the spinal cord of rats treated with hADSC after SCI we used antibodies against pericytes and/or neural cells. Cell within the infiltrates were positive for smooth muscle α-actin (αSMA), a marker for pericytes; Tuj1 and Olig2, markers for neural precursors; and for nestin, vimentin, and NG2, markers for both neural cells and pericytes (Fig. 5,A–F). Cells accumulating in between the two separated vascular basement membranes were negative for Olig2 or Tuj1 and positive for nestin, vimentin, NG2, and αSMA (Fig. 5G–M). Proliferative cells, positive for Ki67 were detected at lesion-associated cell clusters, which correlated with high nestin expression (Fig. 5N–Q). A similar profile of markers expression as well of proliferation was observed after injecting hADSCs into the undamaged spinal cord (Fig. S2), which indicates that the injection of these cells leads to an increased number of neural precursors and/or pericytes in the spinal cord.

### hADSCs Secrete Human Laminin in the Spinal Cord

**Figure 6:**
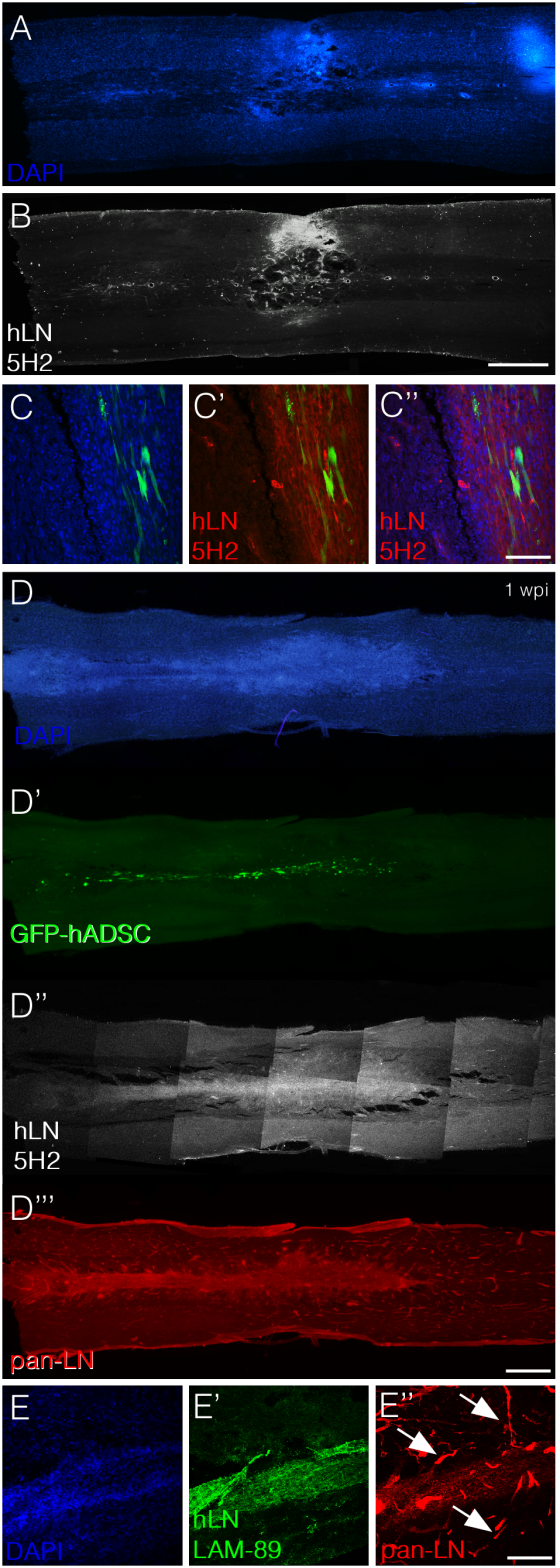
hADSCs secrete laminin in the spinal cord independently of SCI. A–C) Horizontal section of an animal subjected to spinal compression and injected with GFP-hADSCs one week after injury/injection. Panels A and B show photomontages depicting DAPI staining (A) and immunoreactivity for anti-human laminin (B). Note that cell infiltrates and laminin are located in corresponding regions. Panel C shows confocal images demonstrating the coincident localization of GFP-positive hADSCs (green), cell infiltrates (blue) and human laminin (red). D–E) Horizontal section of an uninjured animal one week after transplantation with GFP-hADSCs. Panels D to D’’’ show photomontages depicting DAPI staining (blue, D), GFP-positive hADSCs (D’, green), immunoreactivity for anti-human laminin (D’’, white) and immunoreactivity for anti-pan laminin (D’’’, red). Panel E shows confocal images to demonstrate that while the anti-pan laminin antibody labels rat blood vessels (E’’, red), the anti-human laminin does not (E’). Bars: A, B, D = 1 mm, C = 100 µm, E = 200 µm.

A distinguished feature of niches of neural stem and neural precursors cells in the central nervous system is the presence of the protein laminin in their extracellular matrix [34]–[36]. We therefore investigated if hADSCs would produce laminin *in vivo*, which could be related to the accumulation of neural precursors in the tissue. Using monoclonal antibodies specific for laminin of human origin, we searched for a morphological correlation between areas of high cell density and the deposition of the protein. In a photomontage of a whole horizontal section it is possible to appreciate the high degree of co-localization between cell clusters and laminin deposits both in the injured (Fig. 6A–C) and in the uninjured spinal cord (Fig. 6D–E). Immunostainings for human fibronectin and human collagen IV showed that these two proteins were also present in the cell infiltrates but the deposits were less expressive than those of laminin (Fig. S3).

In order to support the interpretation that laminin associated to cell clusters was produced by hADSCs and not by the rat tissue it was important to confirm the specificity of the anti-human laminin antibodies. We used two different anti-human laminin antibodies to detect the protein. The first one was a mouse monoclonal raised against human laminin (clone LAM-89; Fig. 6E), which is commercially available and described as a reagent that does not cross-react with laminin of rat origin. This monoclonal recognizes the α5 chain and has been extensively used in the literature to label basement membranes in human tissue, particularly those associated to blood vessels [37], [38]. We found no labeling of blood vessels in the rat spinal cord (Fig. 6, compare E’ and E’’). The second antibody was an anti-human α2 (clone 5H2; Fig. 6B–D). To assess the specificity of this antibody we performed immunostainings in rat skeletal muscle, a tissue in which laminin α2 is highly expressed. While a polyclonal anti-pan laminin stained the basement membranes around muscle fibers, monoclonal 5H2 did not recognize the rat muscle tissue (Fig. S4). These results indicate that the laminin matrix associated to the infiltrates of neural precursors was not produced by the rat host but, instead, by the injected human cells.

**Figure 7:**
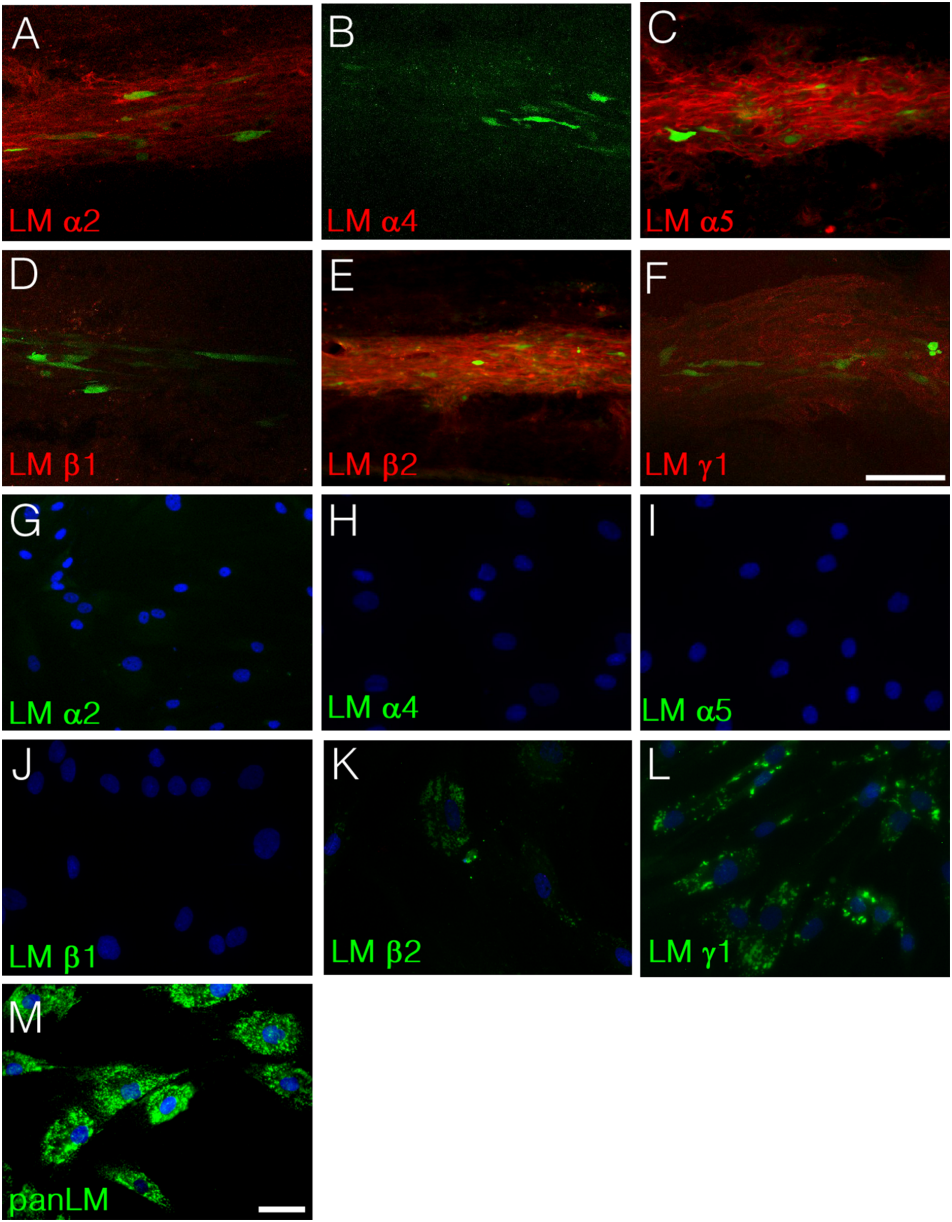
Identification of laminin chains produced by hADSCs *in vivo* and *in vitro*. A–F) Uninjured rats were injected with GFP-hADSCs and the human laminin chains produced in the spinal cord were analyzed one week after transplantation. Confocal images of horizontal sections taken at the spinal midline show that hADSCs are localized in areas rich in human laminin α2 (A), α5 (C), β2 (E) and γ1 (F). Immunoreactivity for human laminin α4 (B) and β1 (D) was not detected in the spinal cord (D). G–M) Analysis of the laminin chains produced by hADSCs cultivated *in vitro*. *In vitro*, the only laminin chains detected were β2 (K) and γ1 (L). Note that hADSCs did not produce α2 (G) nor α5 (I), which suggests that secretion of these two chains is triggered by the contact with the environment of the spinal cord. Panel M shows immunostaining for laminin using an anti-pan laminin antibody. Bars: A–F = 100 µm, G–M = 50 µm.

Results up to here indicate that hADSCs produce laminin α2 and α5 *in vivo*. We next used a panel of anti-human laminin antibodies to investigate which laminin chains appeared in the spinal parenchyma injected with hADSCs. Out of the six isoforms tested, we confirmed expression of α2 and α5 and also found positivity for β2 and γ1 laminin chains (Fig. 7A, C, E, F). The matrix associated to cell clusters was negative for α4 and β1 (Fig. 7B, D). In vitro, hADSCs we did not detect expression of α2, α4, α5 or β1 (Fig. 7G–I). We found positivity only for the β2 and the γ1 chains (Fig. 7K, L) and for anti-pan laminin (Fig. 7M).

**Figure 8:**
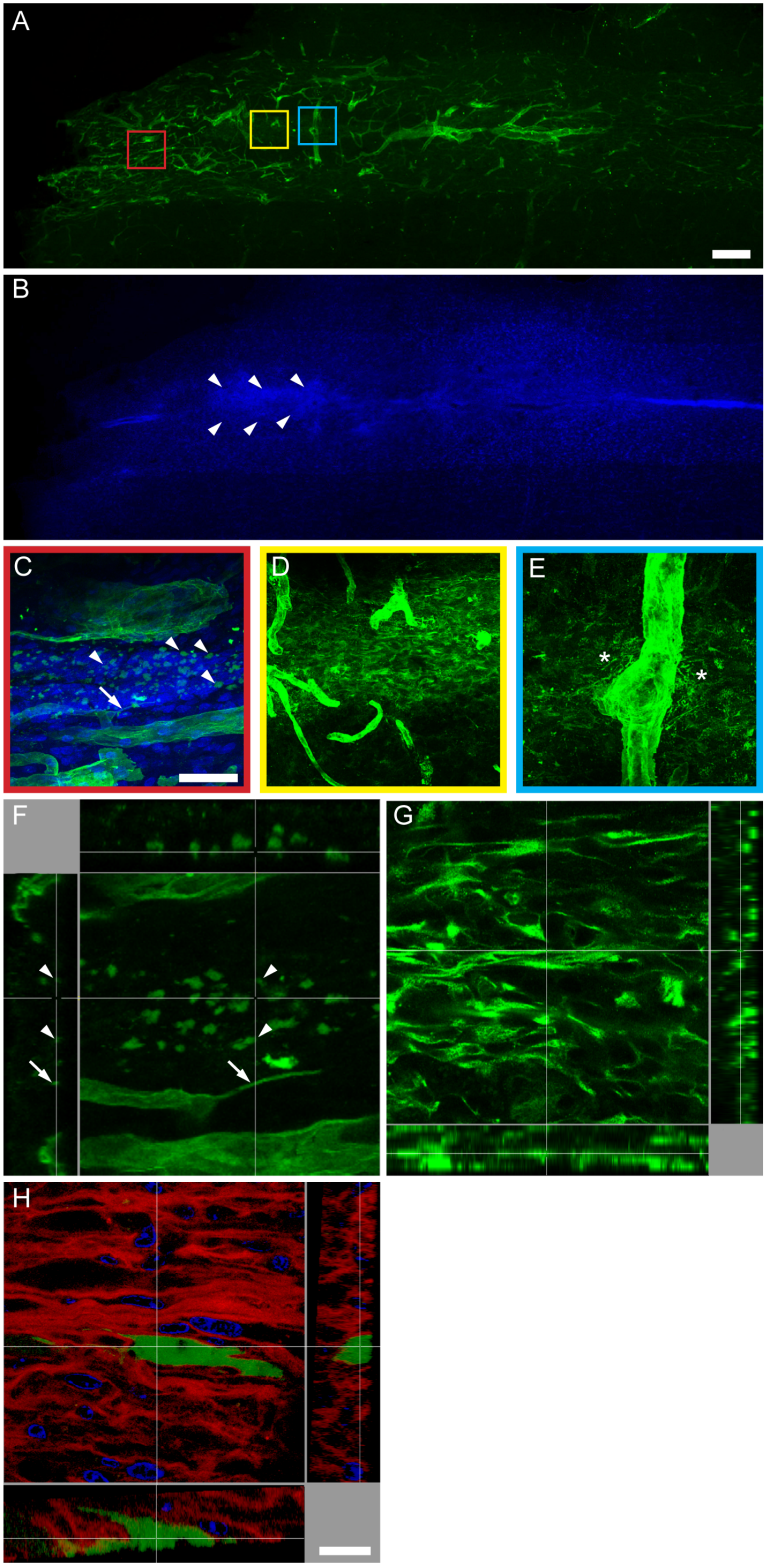
Laminin produced by hADSC forms reticular-like deposits independent of fractones. Photomontage of a horizontal section of the undamaged spinal cord one week after injection of hADSCs (A) immunostained with a pan-specific anti-laminin antibody (green) and (B) counterstained with DAPI (blue). The arrowheads in B delimit the region of the highest increase in cellularity, corresponding to reticular laminin in A. Boxed areas in A were amplified to show the detailed morphology of the types of laminin deposits in the spinal cord. C) The red box shows fractones characterized by the presence of thin lines (arrow) and puncta (arrowhead). D) The yellow box shows an independent reticular-like deposit. E) The blue box shows laminin around a blood vessel. Note that laminin seems to detach from the perivascular basal lamina and to spread in the spinal parenchyma (asterisks). Panels C and D were reconstructed in the z axis to provide a 3D view of the fractones (F) and of the reticular deposits (G). In F, lines (arrow) and puncta (arrowheads) presented similar shapes in 2D or 3D. Panel H shows a 3D view of laminin deposits stained with an antibody specific for the α2 chain of human laminin (red) and counterstained with DAPI (blue). The 3D structure of laminin secreted by hADSCs is similar to the one depicted in G and different from the one depicted in F. Note that DAPI-stained nuclei are nested within reticular laminin (red) and that the protein produced by GFP-transduced hADSCs (green) largely surpasses the borders of the secreting cells.

Based on the recent observation that neurogenic niches in the adult CNS contain a novel type of laminin-rich basal lamina termed fractones [39], we next investigated whether the laminin secreted by hADSCs present the same morphology of fractones. Tissue sections of animals receiving hADSCs into the undamaged spinal cord were immunostained for pan-laminin one week after injection. The polyclonal pan-specific anti-laminin antibody stained all laminin-containing structures present in the tissue. Blood vessels were identified by their overall morphology and diameter above 5 µm (Fig. 8A and E, blue box). Fractones were identified by their organization in puncta with diameter between 1–4 µm and thin lines with 0.1–05 µm width and 5–50 µm length [40] (Fig. 8A and C, red box). In addition, we observed a reticular laminin matrix (Fig. 8A and D, yellow box) similar to the one of human origin secreted by hADSCs (Fig. 6C, E). While a clear increase in cellularity was observed along the whole spinal axis, the location of the most packed cells coincided with the reticular deposit (Fig. 8B, arrowheads). Panels C and D were reconstructed in the z axis to provide views of their 3D organization (Fig. 8F, G). Puncta present in fractones were spherical dense deposits, and the thin lines did not correspond to the side view of sheet-like matrices but instead, resembled essential lines (Fig, 8F). On the other hand, the reticular deposit presented a 3D structure in which each strut was connected to the neighboring one (Fig. 8G). Such reticular mesh is similar to the z-stack obtained using the anti-human α2 laminin antibody (Fig. 8H). DAPI counterstaining showed that cells appearing in the spinal parenchyma seemed trapped into the human laminin mesh secreted by hADSCs (Fig. 8H).

## Discussion

In this study, we showed that treatment with cells isolated from human adipose tissue (hADSCs) improve open field locomotion after a compression injury in rats. We also found that hADSCs secreted human laminin, which accumulates in the spinal cord and increases the presence of neural precursors in the tissue.

The lesion area and the expression of the glial scar marker GFAP were both reduced after treatment with hADSCs, characteristics compatible with a resolutive inflammatory reaction. The treatment also resulted in increased numbers of regenerating fibers. A noteworthy finding was the many 5HT-positive descending axons, particularly at the injury epicenter, observed in hADSC rats as early as one week after lesion. Although the number of fibers decreased at the eighth week after lesion, it still remained much higher than that of non-treated animals. To our knowledge, no studies using cell therapy obtained such a marked improvement in axonal growth as the one promoted by hADSCs in our study.

In the present study, we delivered cells immediately after spinal cord injury. The advantage of this experimental design is that the therapy can contribute to reduce the secondary damage, which initiates within hours after trauma. On the other hand, the disadvantage of the early treatment is that the hemorrhagic and ischemic environment of SCI reduces the survival of the injected cells in the host tissue. Accordingly, the number of GFP-labeled cells progressively decreased between 1 and 7 days after injury and virtually disappeared 8 weeks later. In a previous study, Arboleda and co-workers [26] injected rat ADSCs in immunosuppressed rats one week after injury and reported that living cells remained in the spinal cord during the eight weeks of the experiment. However, these cells did not integrate into the host tissue and their survival did not correlate with the extent of the functional improvements. More recently, Zhou and co-workers [29] reported results compatible with ours, showing that more than 30% of human ADSCs transplanted in immnosuppressed rats survived one week after injection, but that less than 1% remained in the spinal tissue three weeks later. These observations suggest that the regenerative effects of ADSCs were due to the induced secretion of paracrine factors that would stimulate endogenous precursors to regenerate the tissue. It has already been shown that co-culture with BMSCs can prime embryonic stem cells to produce pyramidal neurons able to integrate into the cerebral cortex and to project to the appropriate targets [41], [42]. In addition, a recent study has demonstrated that factors produced by BMSCs can indeed prime neural precursors toward an oligodendrocyte fate [43]. Our results in combination with those of the literature suggest that it is not necessary that injected cells remain in the spinal cord during the regeneration process and that their beneficial priming effects are exerted within the first days after injury.

One important effect of hADSCs was the reduction of the spatial diffusion of macrophage/microglia, restraining these cells to the site of injury. To our knowledge, this is the first time that such an effect has been described as a corollary of cell therapy. It is possible that hADSCs accumulating in the lesion secrete cytokines that attract macrophages to the injury site. Indeed, in agreement with a previous study showing that ADSCs secrete 20 times more IL-6 than BMSCs *in vitro* [44], we observed that hADSCs secrete large amounts of IL-6 in the lesion area (MAN, KM, RB, TC-S, unpublished). Secretion of other cytokines such as IL-10, which is largely produced by hADSCs [45], may also play a role in regeneration, due to its ability to favor the differentiation of M2 macrophages. It has already been shown that BMSCs can shift macrophage differentiation towards the M2 pro-regenerative phenotype [46]–[48].

The present study describes three major novel observations that shed light on the mechanisms of neural tissue regeneration promoted by adult MSC cells. First, hADSCs induced an increase in cellularity at the lesion site and in clusters distributed along the spinal midline above the central canal. Cells in the clusters expressed markers of neural precursors. Likewise, in the non-injured spinal cord progenitor cells accumulated in a single large spindle-like cluster, indicating that the observed increase in cellularity does not depend on the occurrence of a lesion.

In animals receiving hADSCs, the vimentin-positive ependymal cell layer that lines the central canal [49] expanded, indicating that molecules secreted by hADSCs can increase the proliferation of neural precursors in the normal neurogenic area of the spinal cord. Cells from the ependymal layer have already been shown to proliferate after spinal cord injury [50]. This phenomenon could account for the scattered cell clusters seen in the white matter above the canal in animals submitted to injury. In these animals, a second focus of increased cellularity was the lesion epicenter. Progenitor cells could migrate from the neurogenic area of the canal toward the lesion using two alternative routes; directly through the parenchyma or along the perivascular space. Although we cannot presently discriminate between these possibilities, the presence of blood vessels near the expanded lining of the central canal reinforces the latter hypothesis. Similar to our results, it has been previously shown that the injection of bone marrow MSCs stimulated subventricular zone neurogenesis in the mouse brain [51].

The second remarkable feature observed in this study was the appearance of perivascular spaces limited by two clearly definable basement membranes in the spinal cord tissue. Separation of the two basal laminas, which are close together in physiological conditions, creates a perivascular compartment suitable for cell trafficking, associated to inflammation [52], [53]. Recently, it has been proposed that mesenchymal precursors that differentiate into vascular mural cells use conduits in the extracellular matrix called “vascular guiding tunnels” to approach the newly formed endothelial tubes to which they will associate [54].

Cells located between the two membranes expressed nestin, vimentin, and NG2, which are markers for neural precursors and for perivascular cells. They were also positive for αSMA, which is expressed by pericytes of the nervous system [55], but not considered as a marker for neural precursors. This antigenic profile does not allow us to identify these perivascular cells as neural precursors derived from remote neurogenic areas into the lesion site or to perivascular cells with the potential to divide and differentiate into neural lineages. Corroborating the latter, pericytes have recently been proposed to correspond to a heterogeneous population [56] containing multipotent mesenchymal cells with the potential to differentiate into neural precursors [57]. Nevertheless, at this point we cannot determine if neural progenitors originate from the central canal or from the perivascular compartment. Tuj1 and Olig2 expression were not detected in the perivascular compartment, but were detected in the cellular infiltrate at the lesion site and also in the spindle-like cluster in non-damaged spinal cord. Thus, if progenitor cells do in fact migrate through the perivascular compartment they must differentiate into neuroblasts or glial precursors only upon contact with the neural parenchyma.

The third finding providing new information on the mechanisms involved in hADSC-induced regeneration is the accumulation of human laminin in the same areas of the spinal cord where precursor cells have also accumulated. Grafted cells produced a cloud-like pattern of laminin deposition, which corresponded to the location of neural precursors in both injured and non-injured spinal cord. Given that laminins, particularly those containing the α2 and α5 chains [36], are present in CNS regions where neural stem and progenitor cells reside, both during development and in the adult [34], [35], [58], [59], we hypothesize that laminin can be the molecule produced by hADSCs that mediated the accumulation of neural precursors along the midline and at the injury site. In line with this hypothesis is our previous demonstration that acute injection of the protein laminin alone, as long as it is delivered in its polymeric form, promotes regeneration after spinal cord injury [60]. Although in that study we did not search for the presence of neural precursors in the tissue, we did find that laminin treatment led to regeneration of descending axons, to decreases in astrogliosis and cystic cavity size and to an expressive reduction of macrophage spreading in the spinal parenchyma, all events similar to those described here as elicited by hADSC.

This is the first time that a link between laminin and the pro-regenerative effects of mesenchymal cells in the central nervous system is proposed. Previous studies had proposed a connection between laminin and MSCs in other tissues such as the peripheral nerve and skeletal muscle [61], [62]. However, those studies were conducted in laminin deficient animals in which MSCs were injected exactly as an attempt to restore tissue integrity. Furthermore, none of these two studies demonstrated a strong correlation between the localization of endogenous progenitors and laminin deposited by MSC, as shown here.

Bone marrow stromal cells have been shown to express laminin α4, α5, β1, β2 and γ1 [63]. Here we tested whether hADSCs *in vitro* produced these laminin chains, but we found reactivity only for β2 and γ1. In addition, we found no positivity for the α2 chain, which, together with α5, β2 and γ1, was detected *in vivo*. Considering that laminin α2 and α5 of human origin appeared in spinal cord of rats injected with hADSCs (injured or uninjured), we propose that the exposure of the cells to the spinal cord environment was capable of switching the production of laminin chains α2 and α5. These laminin chains secreted only after transplantation of hADSC may stimulate the proliferation and migration of neural precursors from the ependymal layer around the central canal or from other neurogenic niches around blood vessels. The attracted precursor cells accumulate mainly in the lesion site, contributing to regeneration of the nervous tissue and ultimately to functional recovery. In line with this hypothesis, it has previously been shown that laminin isoforms containing the α chains 2, 4 and 5 are essential components of the subependymal neurogenic niche of adult mice [36].

Our results show that hADSCs are efficient in promoting regeneration after SCI and suggest laminin as a mediator of the beneficial effects of these cells. A better understanding of the mechanisms underlying the regenerative effects of stem/progenitor cells in the nervous system is essential for development of future cell-based therapies to treat spinal cord injury in humans.

## Experimental Procedures

### Ethics Statement

All experimental procedures adhered to the guidelines of the American National Institute of Health and the Brazilian COBEA and were approved by the animal welfare committee of the Federal University of Rio de Janeiro/Center of Health Sciences [DAHEICB 041]. All the procedures involving human samples were approved by the Investigational Review Board at HUCFF (protocol numbers 043/09 and 088/04). Bone marrow samples were collected from discharged bone marrow collection kits after aspirates were transferred to infusion bags. Informed consent exemption was approved by the Investigational Review Board at HUCFF since data were analyzed anonymously and derived from discarded samples. Adipose tissue and lipoaspirates were collected after the patients signed a written informed consent.

### Animals

We used adult female Sprague-Dawley rats (200–250 g), bred at the animal facility of the Federal University of Rio de Janeiro. Rats were kept in 12-hour light/dark cycles with free access to food and water in an isolated animal room. Sixty seven rats were used in this study. Forty-eight animals were included in the injured group submitted to spinal cord compression and were further subdivided in cell treated group (n = 27; 3 euthanized at 1 dpi, 12 at 1 wpi, 3 at 4 wpi and 9 at 8 wpi) and vehicle control (n = 21; 3 euthanized at 1 dpi, 6 at 1 wpi, 3 at 4 wpi and 9 at 8 wpi). Non-injured animals that received cells (n = 12; euthanized at 1 wpi) and sham operated animals (n = 6; 3 euthanized at 1 wpi and 3 at 8 wpi) were included. One uninjured animal was injected with human fibroblasts and euthanized one week later.

### Samples and Cells

Fragments of subcutaneous adipose tissue and lipoaspirates were obtained from the abdominal region of patients undergoing plastic surgery at the Clementino Fraga Filho University Hospital (HUCFF), Federal University of Rio de Janeiro, Brazil. Human skin fibroblast cell line was obtained from the Cell Bank of Rio de Janeiro (BCRJ, Rio de Janeiro, RJ, Brazil).

### Plasmid Construct and Lentivirus Production

The pLL3.7 plasmid, originally described by Dr. van Parijs’s group [64], was provided by Dr. Guido Lenz (UFRGS – Porto Alegre, Brazil). Lentiviral vectors were produced as previously described [65], [66].

### Isolation and Transduction of hADSCs

Human adipose tissue-derived stromal cells were obtained as previously described [24], [25], [67]. Briefly, subcutaneous adipose tissue fragments and lipoaspirates were submitted to enzymatic digestion with 10 mg/mL collagenase IA (Sigma-Aldrich, St. Louis, MO) for 1 hour at 37°C under agitation. Cells were plated at 1–2×10^4^ cells/cm^2^ in Dulbecco’s (DMEM Low-glucose, LGC, São Paulo, SP, Brazil) supplemented with 10% fetal bovine serum (FBS, Cultilab, Campinas, SP, Brazil) and antibiotics (100 U/ml of penicillin and 100 µg/ml of streptomycin, both from Sigma-Aldrich) and maintained overnight at 37°C with 5% CO_2_. Non-adherent cells were removed and adherent cells were maintained as above and expanded by enzymatic digestion with 0.125% trypsin and 0.78 mM EDTA (Sigma-Aldrich). Lentivirus transduction was performed by addition of 1 mL of lentiviral vector stock with polybrene at the concentration of 8 µg/mL and 7 mL of DMEM. Cells were then incubated at 5% C0_2_ and 37°C overnight. After incubation, the medium was replaced by fresh DMEM medium with 10% FCS. Cells were kept under these conditions until they were prepared for *in vivo* analysis. The typical multiplicity of infection (MOI) used for ADSC transduction ranged from 10 to 50. Transduced cells were trypsinized, fixed with 4% paraformaldehyde in PBS, and GFP expression was analyzed by flow cytometry (BD FACScalibur Flow Cytometer; Becton Dickinson) using CellQuest software.

### Surgical Procedures

Animals were anesthetized with an i.m. injection (300 µl) of a cocktail containing xylazine (3.2 mg/kg; Syntec, Cotia, SP, Brazil), ketamin (62.5 mg/kg; Syntec), and acepromazin (0.625 mg/kg; Syntec) diluted in water, and were subjected to dorsal laminectomy. For the compression injury, T7 vertebra was removed and a 2-French Fogarty catheter (Baxter Healthcare Corporation, Irvine, CA) was inserted into the dorsal epidural space caudally to T8–T9 spinal level (1 cm distance from the incision). The balloon was inflated with 15 µl of water for 5 min. Rats were kept anesthetized for 30 min before application of acute experimental treatment or control buffers. Soft tissue and skin were sutured in anatomical layers and the animals were allowed to recover in warmed cages and kept in pairs during survival. As specific surgical and post-surgical care, all animals had their eyes lubricated during surgery, received subcutaneous 10 ml injections of Ringer solution for hydration immediately after being sutured, and were treated with gentamicin sulfate (40 mg/kg; Neoquímica, Anápolis, GO, Brazil) for 3 days to avoid urinary infection. The bladder was manually expressed until spontaneous function was reestablished 3 days after injury.

### Spinal Cord Injections

Cell suspensions or vehicle (10 µl) were stereotaxically injected using a 10 µl Hamilton syringe, immediately after injury or after laminectomy in non-injured animals. The total volume was injected once, 1 cm rostrally to the lesion epicenter. In animals, which were not subjected to SCI, injections were made at T8–9 level.

### Histology and Immunofluorescence

Rats were anesthetized and perfused transcardially with 4% paraformaldehyde in 0.1 M phosphate buffer (pH 7.4). Spinal cords were removed and a 1 cm segment between T7–T10 levels was removed. For immunofluorescence analysis, segments were cut in the transversal or horizontal planes in a vibratome (model 3000, Vibratome Co; 40 µm thick), and collected serially onto gelatin-coated glass slides. Tissue samples of gastrocnemius muscle of rats were cut in a cryostat for the anti-human laminin immunolabeling.

Prior to immunolabeling, slides were extensively washed with PBS, permeabilized with 0.3% Triton-X-100 (Sigma), blocked with 10% bovine serum albumin (Sigma) or normal goat serum (Sigma), and finally incubated overnight at 4°C with primary antibodies. The following mouse antibodies were used: anti-GAP-43 (1∶4.000; Sigma); anti-GFAP (1∶500; Sigma); anti-CD68 (ED1; 1∶30; AbD Serotec, Oxford, UK); anti-RECA-1 (1∶100; AbD Serotec), anti-CD3 (clone 1F4; 1∶100; BioLegend, San Diego, CA); anti-αSMA (1∶400; Sigma), anti-class III β-tubulin (Tuj1; 1∶500; Covance, Princeton, NJ), anti-vimentin (1∶100; Sigma), anti-human fibronectin (1∶400; Sigma), anti-human collagen IV (1∶500; Sigma), anti-human laminin α5 chain (clone LAM-89; 1∶1000; Sigma), anti-human laminin α2 chain (clone 5H2; 1∶1000; Millipore), anti-human laminin α2, α4, α5, β1, β2 and γ1, corresponding to clones 5A4, 3D12, 6A11, IIID9, 9F8 and IIID10, were kindly provided by Dr. Lydia Sorokin (University of Muenster, Germany). The following rabbit antibodies were used: anti-serotonin (5HT; 1∶1000; Sigma); anti-Ki67 (1∶100; Abcam); anti-nestin (1∶500; Millipore); anti-Olig2 (1∶500; Millipore); anti-NG2 (1∶150; Millipore); and pan-laminin (polyclonal antibody raised against EHS laminin; 1∶50; Sigma). After rinsing with PBS, sections were incubated for 2 h at room temperature with fluorescent dye-conjugated goat anti-mouse or anti-rabbit IgGs (1∶500; Cy3-conjugated from Sigma and Alexa 488-conjugated from Molecular Probes/Invitrogen). After an additional wash with PBS and one with distilled water, slides were mounted with n-propyl galate (Sigma). In negative controls the primary antibodies were omitted (Fig. S5).

### Image Analysis

Tissue and cells were analyzed in a TE200 fluorescence inverted microscope (Nikon), with standard DAPI/FITC/TRITC filters coupled to a color CCD camera (Evolution vf, Media Cybernetics) and using the software Image Pro Plus 6.0 for image processing (Media Cybernetics Inc.). Images were alternatively acquired with a TCS-SP5 confocal microscope (Leica).

Lesion epicenters and the largest diameter of cavity size were determined by digitalizing the cavity contours of GFAP-stained horizontal sections of the dorsal–ventral plane of the injured cord. The analyses were confined to the middle 640 µm, ranging from 120 to 760 µm below the first section from the top of the spine. For quantification, the section with the largest cavity size was chosen as the epicenter and 5 sections in 5 successive slides on each side of this putative epicenter had their areas calculated using the software Image Pro Plus 6.0 (Media Cybernetics Inc.) and plotted against a spatial axis. We evaluated three animals of each experimental group.

GAP-43 and 5-HT fibers were evaluated in conventional fluorescence images and plotted as the sum of the total length of positive fibers, at 3 different regions (injury epicenter; 500 µm rostral and caudal to epicenter), in 3 animals (1 section per animal).

### Behavioral Assessment

Locomotor activity was evaluated using the open-field walking test, BBB [68]. One animal at a time was allowed to move freely inside a circular plastic tray for 5 min, and two independent examiners who were blinded to the experiment attributed scores. The final score of each animal was the mean value of both examiners.

### Statistics

Comparisons between different experimental groups were performed using GraphPad Prism version 5.00 for Windows (GraphPad Software, San Diego, USA) to analyze the variance (ANOVA) with post-hoc Kruskal-Wallis test for multiple comparisons (BBB assays). For comparison between 2 groups we used the Student’s t test (cavity size, ED1, GAP-43, and 5-HT labeling). Differences were considered significant when p<0.05.

## Supporting Information

Figure S1 **Neighboring blood vessels interact with the expanded wall of the central canal.** (A–F) Consecutive confocal images in the z axis of a horizontal section at the level of the central canal processed for DAPI staining (blue) 1 week after injury. It is possible to see the transition between a single cell (A) and a multiple cell (B) lining of the canal wall (dashed line); the progressive thickening of the canal wall (D–E); a blood vessel approaching the canal area (arrows in B–F); and cells from the canal wall contacting the lumen of this blood vessel (F). (G–I) Higher magnification images of boxed areas in D–F, showing the area of interaction between the blood vessel and the central canal with high cell density. GFAP immunoreactivity (red) reveals the areas of tissue as opposed to hollow structures such as the blood vessel and the canal. Dashed lines delineate the border between the wall of the central canal and the spinal grey matter. Bars: A–F = 50 µm and G–I = 25 µm.
(TIF)

Figure S2 **Areas of increased cellularity in the uninjured spinal cord injected with hADSC contain neural precursors and/or pericytes.** A–C) Confocal images of a transverse section of the spinal cord immunostained with anti-CD68 (ED1, red), one week after the injection of GFP-hADSC into the undamaged spinal cord. Note that only a few scattered cells correspond to macrophages/microglial cells appearing in cell infiltrates (DAPI, blue). D–S) Confocal images of horizontal sections of the undamaged spinal cord one week after injection of hADSCs, showing the presence of neural precursors and/or pericytes, identified by immunostaining with anti-nestin (D–G, R, S, green), anti-vimentin (H, I, red; J, K, green), Tuj1 (L,M, red), anti-SMA (N,O, red) and anti-Olig2 (P, Q, green). Note that the phenomenon of separation of the two laminas around blood vessels also occurs in the absence of lesion (F, G, J, K). Nestin-positive cells (green) present Ki67-positive nuclei (red), indicating that neural precursors proliferate in the cell infiltrates (R, S). Bars: A, H, I, P, Q = 100 µm; B, C, F–I, K, S = 25 µm; D, E, L–O, R: = 50 µm.
(TIF)

Figure S3 **Extracellular matrix proteins secreted by the hADSCs in the undamaged spinal cord.** Confocal images of horizontal sections of the spinal cord, one week after injection of GFP-hADSCs (green). Images show immunoreactivity for anti-human fibronectin (B, C, red), anti-human collagen IV (E, F, red) and anti-human laminin (clone 5H2, H, I, red) counterstained with DAPI (blue) to reveal cell infiltrates (A, D, G). Note that human laminin is more abundant than the other proteins. Bars: A–I = 100 µm.
(TIF)

Figure S4 **The anti-human α2 laminin antibody does not cross-react with rat α2 laminin.** The specificity of the anti-human laminin α2 antibody (clone 5H2) was investigated by testing its ability to recognize the rat muscle, where α2 is the major component of the basal lamina. Rat gastrocnemius muscle was cut transversally and stained with an anti-pan laminin antibody (A) or with 5H2 (B). Note that basement membranes around muscle fibers were labeled by anti-pan but not by anti-human α2 laminin. A negative control obtained by omitting the primary antibodies is shown in panel C. DAPI counterstaining appears in panels D–F. Scale bar = 100 µm.
(TIF)

Figure S5 **Negative controls for immunolabeling analyses.** A–F) Confocal images of horizontal sections of the spinal cord incubated with fluorescent dye-conjugated goat Cy3-anti-rabbit (A, red), Alexa 488-anti-mouse (B, green), Cy3-anti-mouse (D, red), Alexa 488-anti-rabbit (E, green). Red and green channels are shown superimposed (C, F) together with DAPI counterstaining (blue). Bars: A–F = 200 µm.
(TIFF)

Movie S1 **Cells accumulate in between the two laminin-rich basement membranes around blood vessels in the spinal cord of hADSC-treated animals.** The animation was generated from a series of confocal optical slices of a horizontal section (50 µm thick) of the rat spinal cord 1 week after injury and injection of hADSC. The slice was immunostained for PAN-laminin (red) and nestin (green) and counterstained with DAPI (blue). Cells tend to accumulate in nestin-rich areas both between the two basement membranes and in the spinal parenchyma. Note that in the inferior part of the image cells seem to escape from the vessel in a region where the laminin membrane appears disrupted. Confocal micrographs were analyzed using a free trial version of Imaris software (version 7.2, Bitplane Scientific Software).
(AVI)
