# Supplementary figures and images for: Human Mesenchymal Cells from Adipose Tissue Deposit Laminin and Promote Regeneration of Injured Spinal Cord in Rats
Source: PLoS One. 2014 May 15;9(5):e96020. doi: 10.1371/journal.pone.0096020 (PMC4022508; doi:10.1371/journal.pone.0096020)

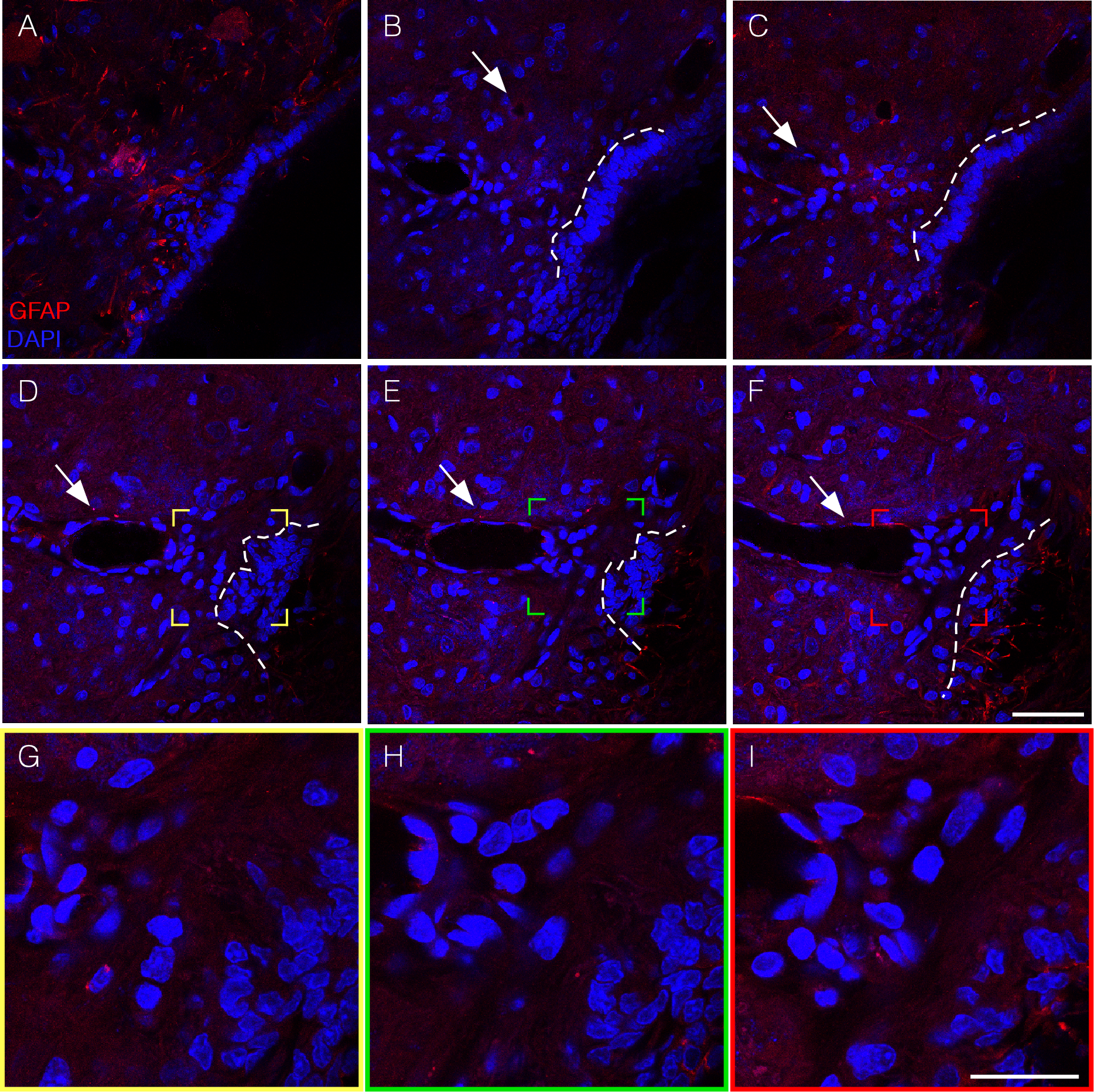

Supplement: Figure S1 — Neighboring blood vessels interact with the expanded wall of the central canal. (A–F) Consecutive confocal images in the z axis of a horizontal section at the level of the central canal processed for DAPI staining (blue) 1 week after injury. It is possible to see the transition between a single cell (A) and a multiple cell (B) lining of the canal wall (dashed line); the progressive thickening of the canal wall (D–E); a blood vessel approaching the canal area (arrows in B–F); and cells from the canal wall contacting the lumen of this blood vessel (F). (G–I) Higher magnification images of boxed areas in D–F, showing the area of interaction between the blood vessel and the central canal with high cell density. GFAP immunoreactivity (red) reveals the areas of tissue as opposed to hollow structures such as the blood vessel and the canal. Dashed lines delineate the border between the wall of the central canal and the spinal grey matter. Bars: A–F = 50 µm and G–I = 25 µm. (TIF) [file pone.0096020.s001.tif]

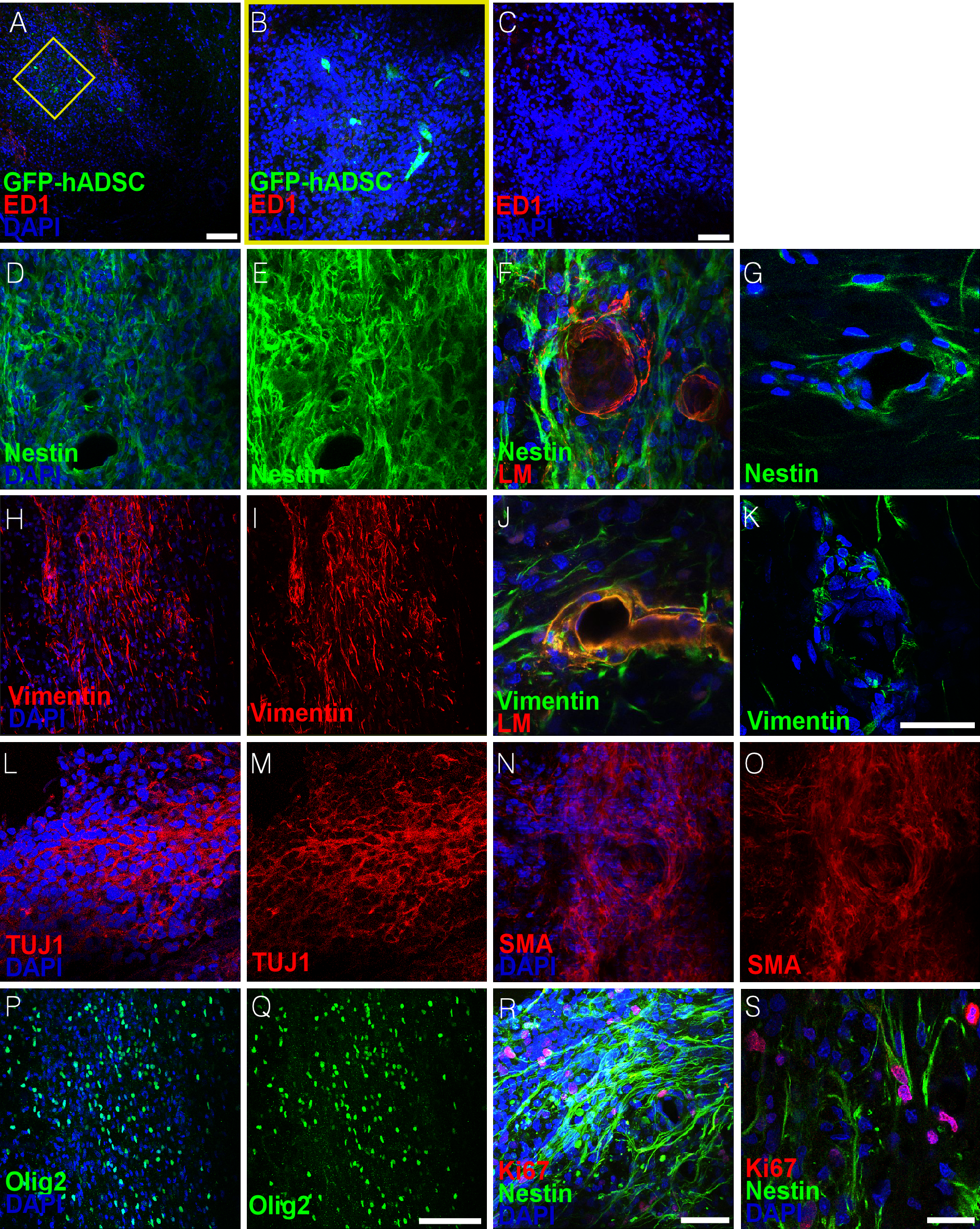

Supplement: Figure S2 — Areas of increased cellularity in the uninjured spinal cord injected with hADSC contain neural precursors and/or pericytes. A–C) Confocal images of a transverse section of the spinal cord immunostained with anti-CD68 (ED1, red), one week after the injection of GFP-hADSC into the undamaged spinal cord. Note that only a few scattered cells correspond to macrophages/microglial cells appearing in cell infiltrates (DAPI, blue). D–S) Confocal images of horizontal sections of the undamaged spinal cord one week after injection of hADSCs, showing the presence of neural precursors and/or pericytes, identified by immunostaining with anti-nestin (D–G, R, S, green), anti-vimentin (H, I, red; J, K, green), Tuj1 (L,M, red), anti-SMA (N,O, red) and anti-Olig2 (P, Q, green). Note that the phenomenon of separation of the two laminas around blood vessels also occurs in the absence of lesion (F, G, J, K). Nestin-positive cells (green) present Ki67-positive nuclei (red), indicating that neural precursors proliferate in the cell infiltrates (R, S). Bars: A, H, I, P, Q = 100 µm; B, C, F–I, K, S = 25 µm; D, E, L–O, R: = 50 µm. (TIF) [file pone.0096020.s002.tif]

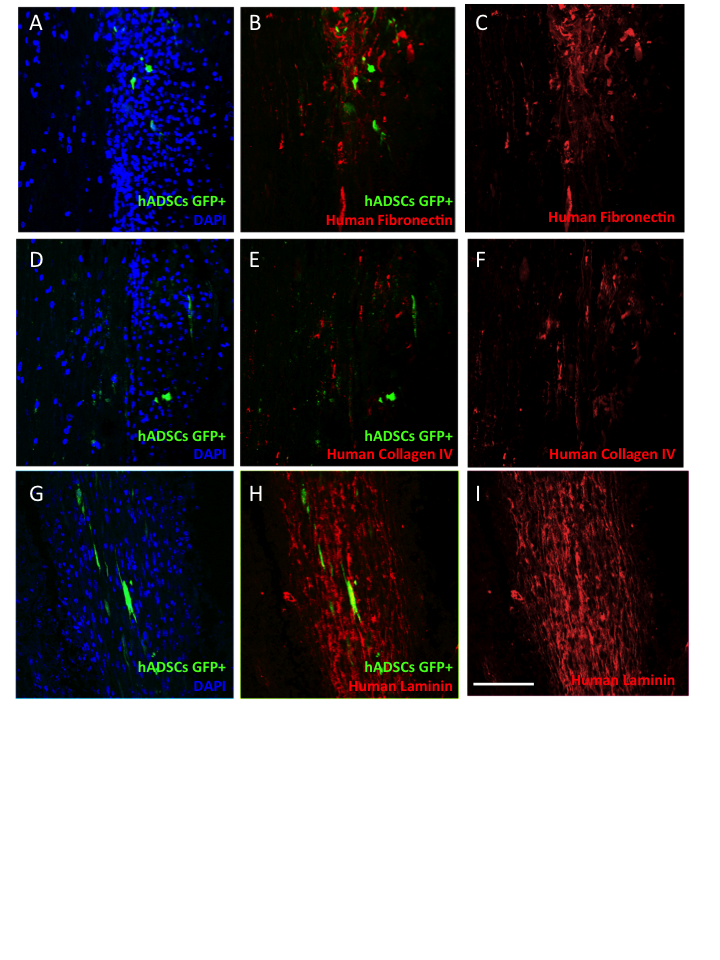

Supplement: Figure S3 — Extracellular matrix proteins secreted by the hADSCs in the undamaged spinal cord. Confocal images of horizontal sections of the spinal cord, one week after injection of GFP-hADSCs (green). Images show immunoreactivity for anti-human fibronectin (B, C, red), anti-human collagen IV (E, F, red) and anti-human laminin (clone 5H2, H, I, red) counterstained with DAPI (blue) to reveal cell infiltrates (A, D, G). Note that human laminin is more abundant than the other proteins. Bars: A–I = 100 µm. (TIF) [file pone.0096020.s003.tif]

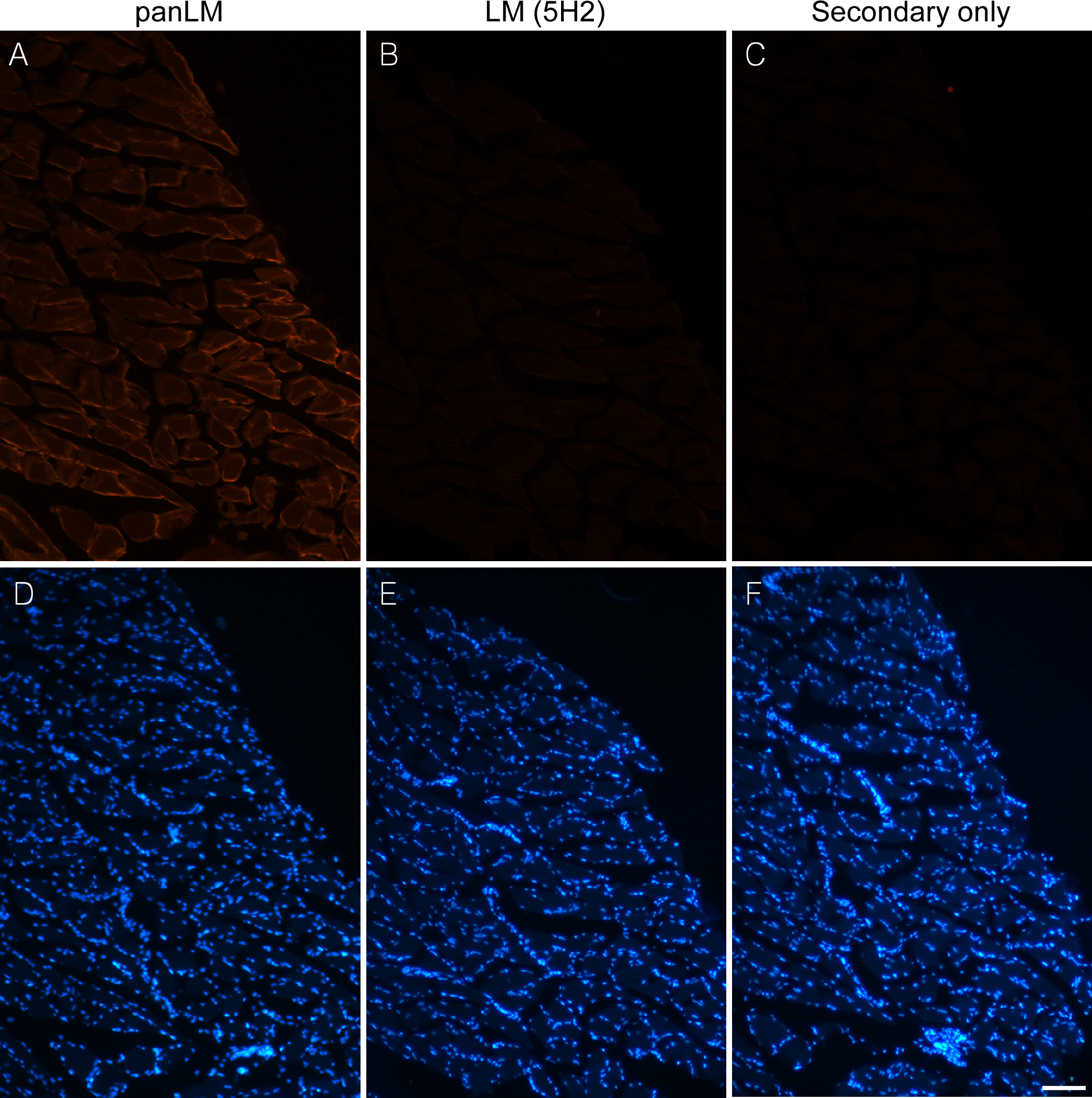

Supplement: Figure S4 — The anti-human α2 laminin antibody does not cross-react with rat α2 laminin. The specificity of the anti-human laminin α2 antibody (clone 5H2) was investigated by testing its ability to recognize the rat muscle, where α2 is the major component of the basal lamina. Rat gastrocnemius muscle was cut transversally and stained with an anti-pan laminin antibody (A) or with 5H2 (B). Note that basement membranes around muscle fibers were labeled by anti-pan but not by anti-human α2 laminin. A negative control obtained by omitting the primary antibodies is shown in panel C. DAPI counterstaining appears in panels D–F. Scale bar = 100 µm. (TIF) [file pone.0096020.s004.tif]

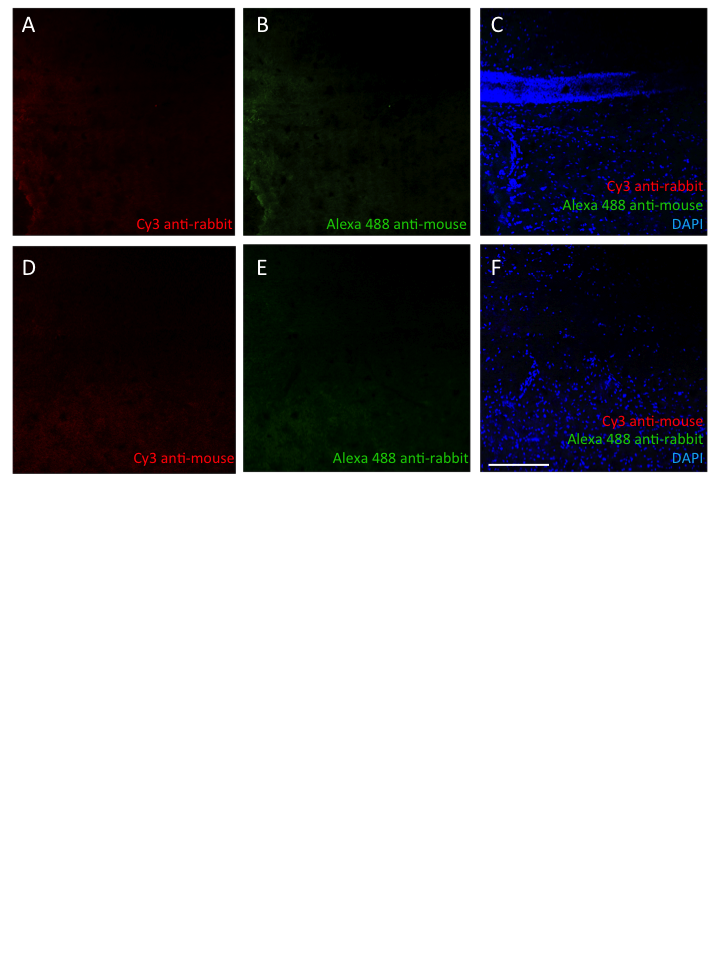

Supplement: Figure S5 — Negative controls for immunolabeling analyses. A–F) Confocal images of horizontal sections of the spinal cord incubated with fluorescent dye-conjugated goat Cy3-anti-rabbit (A, red), Alexa 488-anti-mouse (B, green), Cy3-anti-mouse (D, red), Alexa 488-anti-rabbit (E, green). Red and green channels are shown superimposed (C, F) together with DAPI counterstaining (blue). Bars: A–F = 200 µm. (TIFF) [file pone.0096020.s005.tiff]
